# High-level secretion of native recombinant human calreticulin in yeast

**DOI:** 10.1186/s12934-015-0356-8

**Published:** 2015-10-15

**Authors:** Evaldas Čiplys, Eimantas Žitkus, Leslie I. Gold, Julien Daubriac, Savvas C. Pavlides, Peter Højrup, Gunnar Houen, Wen-An Wang, Marek Michalak, Rimantas Slibinskas

**Affiliations:** Department of Eukaryote Gene Engineering, Institute of Biotechnology, Vilnius University, V.A. Graičiūno 8, 02241 Vilnius, Lithuania; Division of Translational Medicine, Department of Medicine, New York University School of Medicine, 550 First Avenue, NB17E4, New York, NY 10016 USA; Department of Biochemistry and Molecular Biology, University of Southern Denmark, Campusvej 55, 5230 Odense, Denmark; Department of Autoimmunology and Biomarkers, Statens Serum Institut, Artillerivej 5, 2300 Copenhagen, Denmark; Department of Biochemistry, University of Alberta, Edmonton, AB T6G 2H7 Canada

## Abstract

**Background:**

Calreticulin (CRT) resides in the endoplasmic reticulum (ER) and functions to chaperone proteins, ensuring proper folding, and intracellular Ca^2+^ homeostasis. Emerging evidence shows that CRT is a multifunctional protein with significant roles in physiological and pathological processes with presence both inside and outside of the ER, including the cell surface and extracellular space. These recent findings suggest the possible use of this ER chaperone in development of new therapeutic pharmaceuticals. Our study was focused on human CRT production in two yeast species, *Saccharomyces cerevisiae* and *Pichia pastoris*.

**Results:**

Expression of a full-length human CRT precursor including its native signal sequence resulted in high-level secretion of mature recombinant protein into the culture medium by both *S. cerevisiae* and *P. pastoris*. To ensure the structural and functional quality of the yeast-derived CRTs, we compared yeast-secreted human recombinant CRT with native CRT isolated from human placenta. In ESI–MS (electrospray ionization mass spectrometry), both native and recombinant full-length CRT showed an identical molecular weight (mass) of 46,466 Da and were monomeric by non-denaturing PAGE. Moreover, limited trypsin digestion yielded identical fragment patterns of calcium-binding recombinant and native CRT suggesting that the yeast-derived CRT was correctly folded. Furthermore, both native and recombinant CRT induced cellular proliferation (MTS assay) and migration of human dermal fibroblasts (in vitro wound healing assay) with the same specific activities (peak responses at 1–10 ng/ml) indicating that the functional integrity of yeast-derived CRT was completely preserved. Simple one-step purification of CRT from shake-flask cultures resulted in highly pure recombinant CRT protein with yields reaching 75 % of total secreted protein and with production levels of 60 and 200 mg/l from *S. cerevisiae* and *P. pastoris*, respectively. Finally, cultivation of *P. pastoris* in a bioreactor yielded CRT secretion titer to exceed 1.5 g/l of culture medium.

**Conclusions:**

Yeasts are able to correctly process and secrete large amounts of mature recombinant human CRT equally and fully biologically active as native human CRT. This allows efficient production of high-quality CRT protein in grams per liter scale.

**Electronic supplementary material:**

The online version of this article (doi:10.1186/s12934-015-0356-8) contains supplementary material, which is available to authorized users.

## Background

CRT functions in the ER as a calcium-binding chaperone involved in a variety of biological processes including quality control of protein folding [1–3], regulation of calcium homeostasis [2, 4, 5] and MHC class I antigen processing [6, 7]. In addition to these essential intracellular functions, CRT has other important functional roles outside of the ER that are critical to various physiological and disease processes, particularly stress-related disease [8]. For example, CRT has a significant role in both the innate and adaptive immune response and cell surface CRT is required for phagocytosis of apoptotic cells [9]. In this vein, both intracellular and extracellular CRT are important in the host immune response to cancer with respect to activation of T cells, peptide loading with tumor antigens, and in the phagocytosis of tumor cells expressing cell surface CRT, by dendritic cells. CRT is also important to the success of certain chemotherapies [10–12]. In addition, CRT is integrally important in the process of healing cutaneous wounds [13, 14]. These functions are directed by the surface-exposed or secreted protein form of CRT [14, 15]. The translocation of CRT to the cell surface can be induced by ER stress in some cell types, which is triggered by various stimuli including anthracyclines, irradiation and reduction of ER Ca^2+^ levels [16–18]. Importantly, since exogenous CRT rescues CRT-deficient cells in numerous and different CRT-dependent functions, such as adhesion, migration, phagocytosis, and immunoregulation [8], exogenously supplied CRT has significant therapeutic potential for a variety of indications including impaired diabetic wound healing and cancer therapy [8, 10, 11, 13, 14, 19]. In fact, chronic wound healing and particularly impaired cutaneous healing as a consequence of diabetes is a global serious unmet medical need and economic challenge (26 million worldwide patients). Taken together, future fundamental, applied and therapeutic studies and use will likely require large amounts of affordable high-quality recombinant human CRT protein with native functional capacity, insofar as possible.

Here we show that expression of a full-length human CRT precursor in yeast cells can be employed to obtain a high level of secretion of mature native recombinant CRT protein. Fed-batch fermentation of *P. pastoris* culture resulted in more than 1.5 g/l CRT secretion yield. Comparison of yeast-secreted recombinant CRT to native CRT isolated from human placenta showed that the recombinant protein has apparent similar molecular and functional properties as the native protein. From this study, we can conclude that yeast is an excellent host for industrially relevant production of native recombinant CRT protein.

## Results and discussion

### Expression and purification of human CRT from yeasts *S. cerevisiae* and *P. pastoris* from culture medium

**Fig. 1:**
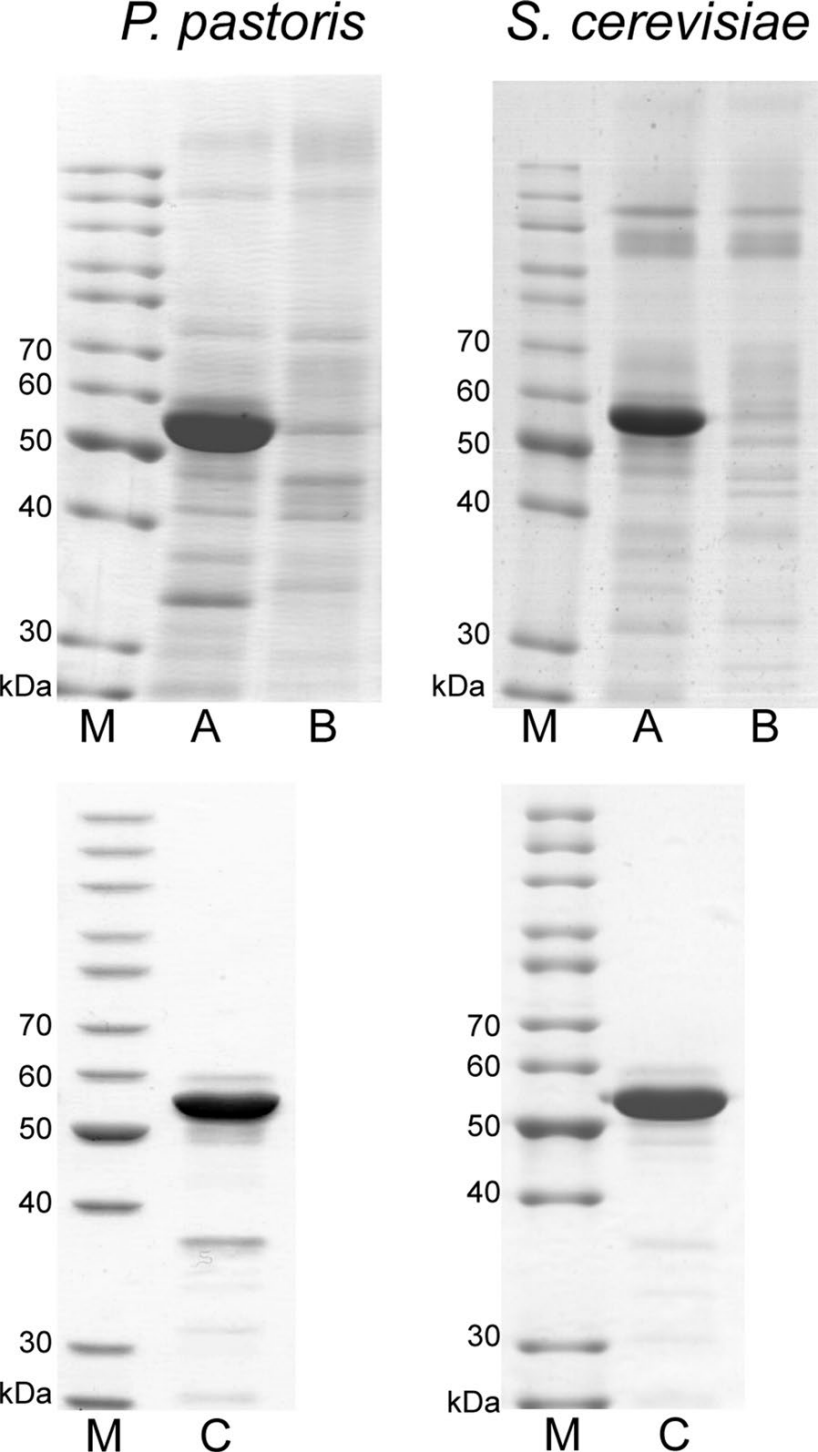
SDS-PAGE analysis of yeast culture media and purified recombinant human CRT samples. *Upper panel* yeast culture media after CRT expression in *P. pastoris* and *S. cerevisiae* (10× concentrated media supernatants). *A* media from yeast strains expressing human CRT. *B* media from control yeast cells without *CRT* gene. *Lower panel* purified secreted recombinant human CRT protein (*C* *lanes*). *M* unstained protein ladder (ThermoScientific, cat. no. 26614)

Our results herein show that expression of a full-length human CRT precursor including its native signal sequence resulted in a high-level secretion of the recombinant protein into the culture medium by two yeast species, *S. cerevisiae* and *P. pastoris*. It should be noted that the expressed human CRT polypeptide precursor contained exactly the same native amino acid sequence as CRT precursor in human cells. Neither yeast secretion signal sequences, nor any tags for purification were used in this study. In the *S. cerevisiae* system, we expressed the gene encoding CRT under control of the yeast *PGK1* gene promoter in the same vector, pFDC [20], that was previously used for the expression of secreted human BiP and ERp57 in yeast [21, 22] and show that CRT was similarly secreted into the culture medium (Fig. 1). However, quantities of the secreted CRT protein were much higher than previously observed for human BiP or ERp57. According to data obtained from densitometric analysis of SDS–PAGE gels, secreted human recombinant CRT constituted approximately 60–65 % of the total *S. cerevisiae* secreted proteins. The CRT secretion titer was approximately 60-65 mg from 1 L of yeast culture medium. The amount of secreted CRT in *S. cerevisiae* culture supernatant was about four- to fivefold higher than either human BiP or ERp57 under the same conditions (for direct comparison of secretion levels see SDS-PAGE gel in Additional file 1). CRT secretion was also achieved by using methanol-inducible yeast *AOX1* gene promoter in *P. pastoris* (Fig. 1). Shake flask cultures of the selected *P. pastoris* transformant, CPp9, with multicopy integrations of CRT expression cassettes yielded approximately 180–200 mg/l of secreted CRT and the purity of the full-length mature human CRT recombinant protein in crude *P. pastoris* culture medium was approximately 70–75 %. There were also a few minor bands of partially degraded CRT protein detected in the culture media from both yeasts by SDS-PAGE and Western blot, but these bands were not included in calculations.

**Fig. 2:**
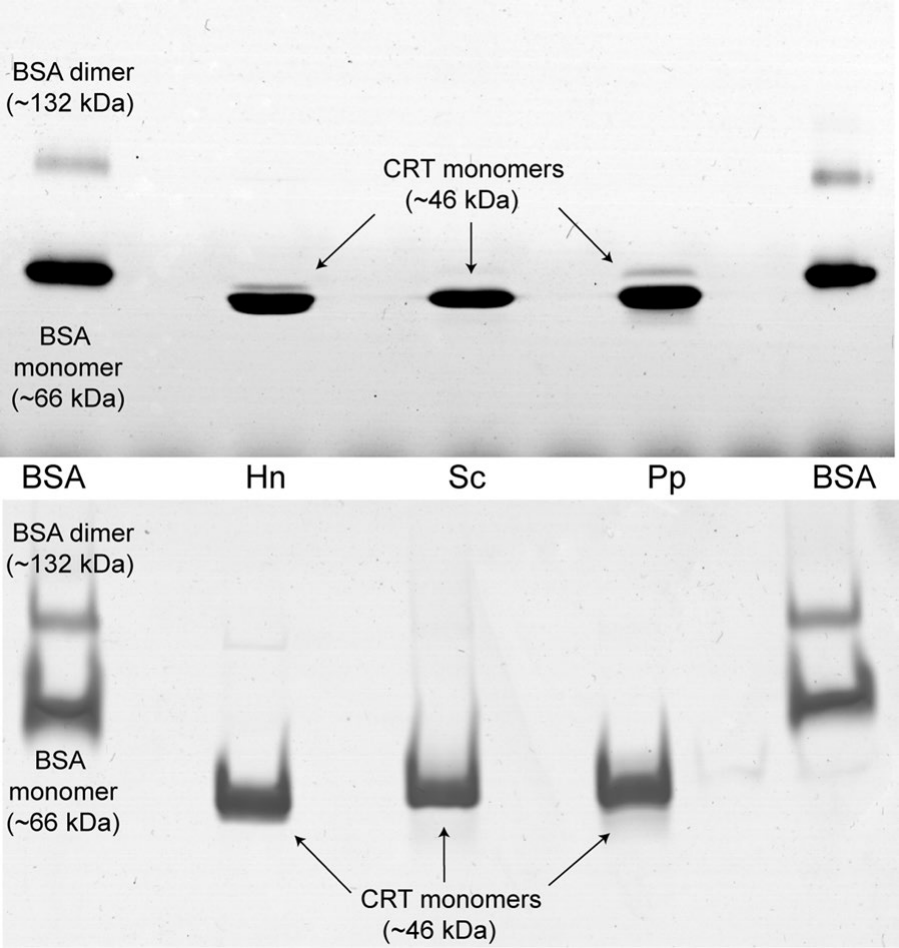
Native PAGE of human placental and recombinant CRTs. Blue native PAGE is shown above and native PAGE below. 5 μg of purified CRT was loaded onto each gel lane. Protein source is indicated at the lanes as follows: *Hn* native CRT from human placenta; *Sc* recombinant secreted human CRT from *S. cerevisiae*; *Pp* recombinant secreted human CRT from *P. pastoris*. *BSA* was loaded as molecular weight marker

The high-level secretion of recombinant human CRT in yeast culture media allowed one-step purification by Q-Sepharose ion-exchange chromatography. CRT is an acidic protein with a pI of 4.29 and contains an Asp/Glu/Lys-rich C-domain. Such biophysical characteristics allow strong binding to an anion-exchange column even at low pH and increased ionic strength. This purification step efficiently removed yeast proteins and other impurities. However, lower molecular weight (MW) forms of CRT were co-purified with the main form of the full-length CRT protein in the same single elution peak (Additional file 2). There was also a slightly higher MW protein form detected by SDS-PAGE in elution fractions of purified CRT from both yeasts, which was more prominent in *P. pastoris*-derived preparation (Additional file 2b, d). The elution profile showed that the CRT peak was not symmetrical and even had a smaller shoulder in preparation from *P. pastoris* (Additional file 2c), possibly due to presence of additional minor protein forms observed. Western blotting analysis showed that minor bands visible by SDS-PAGE of purified protein also represent CRT as they reacted with monoclonal anti-CRT antibody (Additional file 3). It was also evident that CRT fragments of lower MW were generated during biosynthesis, because they were also observed in yeast culture medium (Fig. 1; Additional file 3). Moreover, we did not observe such multiple fragments of lower MW by native PAGE (Fig. 2). It seems that after proteolytic cleavage lower MW fragments are still maintained within CRT molecules by intra-molecular interactions, while they are separated only under denaturing conditions. Probably, such protein form should be considered as “nicked”, rather than “partially degraded”. SDS-PAGE and immunoblotting showed the same overall pattern of “nicked” or partially degraded forms between different preparations (Additional file 4). According to densitometric analysis of Coomassie-stained SDS–PAGE gels, the major intact CRT protein form constituted for approximately 80 % in *P. pastoris*- and 85 % in *S. cerevisiae*-derived preparations. The overall purity of CRT including minor protein forms was considerably higher than 90 % in preparations from both yeasts. Furthermore, recombinant CRT purified from *P. pastoris*, analysed for protein impurities by quantitative evaluation of tryptic peptides using a HDMS Synapt G2 mass spectrometer, showed 98–99 % purity. Therefore, this simple one-step purification generated highly pure recombinant secreted human CRT containing minor amounts of nicked or partially degraded protein forms. The yields of purified CRT were 45–50 and 130–150 mg/l in *S. cerevisiae* and *P. pastoris*, respectively.

### Recombinant human CRT secreted by yeast and CRT protein isolated from human placenta have similar molecular features

**Fig. 3:**
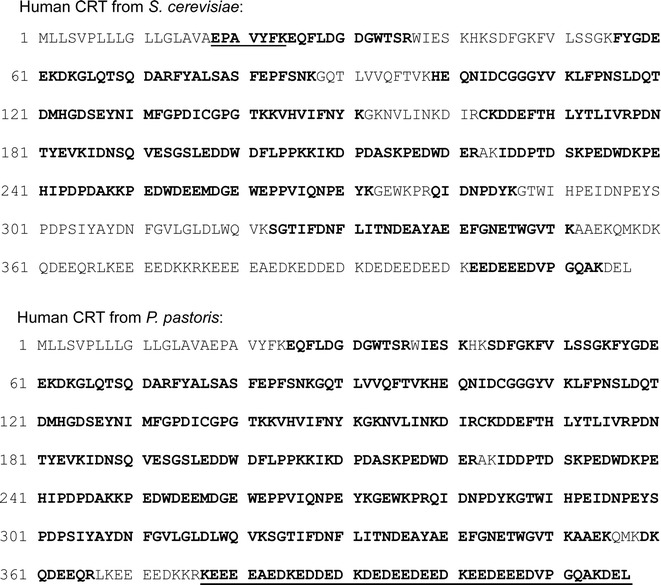
Tryptic peptide mass fingerprinting of yeast-secreted CRT protein. Purified secreted CRT protein was analyzed by trypsin digestion and MALDI-TOF/TOF tandem MS/MS (*upper panels*) or nano-LC coupled with HDMS Synapt G2 mass spectrometer (*lower panels*). Human CRT was identified with ~66 % or ~91 % sequence coverage in *S. cerevisiae*- or *P. pastoris*-derived samples, respectively (identified sequences are indicated in *bold*). The N-terminal tryptic peptide (begins not from lysine or arginine), and C-terminal tryptic peptide (contains C-terminal -KDEL sequence), are *underlined*

The pattern from tryptic peptide mass fingerprinting of purified secreted CRT from both yeast species was confirmed to be the same as mature CRT sequence from human cells (without the signal sequence) including a tryptic peptide (EPAVYFK) corresponding to the N-terminal sequence of native mature CRT [23–25] (Fig. 3). Moreover, using a comprehensive technique with a HDMS Synapt G2 system, peptide mass fingerprinting of *P. pastoris*-secreted CRT was able to provide >90 % amino acid sequence coverage (Fig. 3). Although we were not able to identify the N-terminal tryptic peptide by this method, a C-terminal human CRT peptide including the intact ER retention/retrieval signal KDEL was identified (underlined in Fig. 3).

**Fig. 4:**
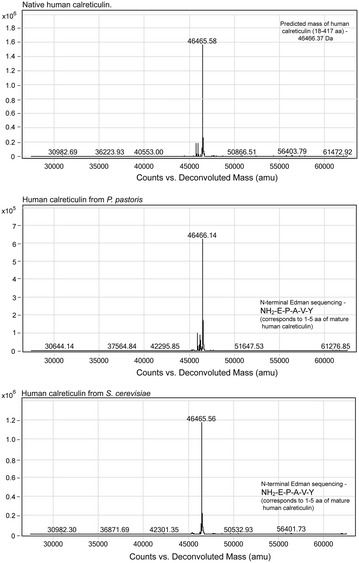
Deconvoluted ESI–MS spectra of native and recombinant human CRTs. Analyzed proteins were purified from human placenta (native CRT) or from culture media of yeast *P. pastoris* and *S. cerevisiae*, respectively. Results of N-terminal Edman sequencing are shown as inserts for secreted recombinant CRTs

To ensure the structural and functional integrity of the yeast-derived CRTs, we compared yeast-secreted human recombinant CRT with native CRT isolated from human placenta [26]. ESI–MS of a whole intact native and recombinant CRTs showed basically the same molecular weight (MW) of approximately 46,466 Da, which exactly corresponds to the theoretically calculated MW of mature human CRT (Fig. 4). Moreover, N-terminal sequencing by Edman degradation confirmed that the first five N-terminal amino acid sequences of *P. pastoris*- and *S. cerevisiae*-expressed proteins were NH_2_-EPAVY and corresponded to the N-terminal amino acid sequence of mature human CRT. Taken together, these results indicated that native CRT human ER signal sequence was recognized and correctly processed (i.e., cleaved from the precursor to form the mature protein) in yeast cells, and this supported translocation of recombinant protein into the ER lumen followed by secretion into the culture media. Furthermore, according to the determined MW, both native and recombinant CRTs are pure polypeptide chains without additional groups attached due to post-translational modification. The lack of such post-translational modifications as glycosylation and phosphorylation, was shown in previous analysis of human placental CRT [26]. Nevertheless, it should be noted that our study was focused on comparison of CRTs from different sources, rather than on analysis of possible post-translational modifications. The protein should contain a disulfide bond [26] and there were reports on various CRT modifications including acetylation of lysine residues [27, 28], glycosylation [29] and citrullination [30] in different tissues or under specific conditions. Some modification of both recombinant and native placental CRTs might be suspected from a minor form of slightly higher MW observed in SDS-PAGE, as it was mentioned above. According to densitometric scanning of SDS-PAGE gels it constituted up to 2 % of protein purified from *P. pastoris* or human placenta, and even less amount of protein from *S. cerevisiae*. This minor band should represent the same CRT protein as it reacted with anti-CRT antibodies (Additional files 1, 3 for *S. cerevisiae* and *P. pastoris*, respectively). However, both minor and major bands have been characterised by MS and no differences were seen. The most likely explanation may be that the slower migrating band represents a separate conformation, which is stable during the time of electrophoresis, possibly proline cis–trans isomerism (CRT has many prolines). At least, they certainly do not represent acetylation or glycosylation. Although we did not perform extensive tests for modifications and can thus not exclude that some modifications may still exist, the methods used in this study did not detect any difference in modification status between native and recombinant CRTs. Taken together, based on the analyses herein, the native and recombinant CRT proteins appear to show similar molecular characteristics.

### Oligomerization and conformation of yeast-secreted and placental CRTs

Oligomerization analysis by native and blue native PAGE showed that both placental and recombinant CRTs were present only in monomeric form (Fig. 2). However, we observed a slight difference in mobility of CRTs purified from human placenta and yeast culture media. Native human protein migrated slightly faster than recombinant CRTs, and this suggested a conformational difference. In order to check possible influence of different buffers used for storage of recombinant and placental CRTs, we have transferred the latter to storage buffer of recombinant CRTs and repeated both native PAGEs. Indeed, buffer change resulted in slower migration of placental CRT and it became very similar to that of yeast-secreted proteins (Additional file 5).

**Fig. 5:**
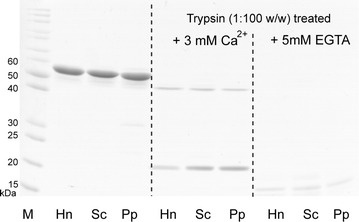
Partial digestion of native and recombinant human CRTs with trypsin. *M* unstained protein ladder (ThermoScientific, cat. no. 26614). Undigested CRTs are loaded to the *left*, CRTs digested with trypsin in the presence of 3 mM CaCl_2_—in the *middle*, whereas CRTs digested with trypsin after addition of EGTA to chelate calcium ions—in the *right gel lanes*. Source of protein is indicated as described in Fig. 2 legend

Conformation of native and recombinant CRTs was analysed by limited proteolysis with trypsin. It has been reported that the binding of Ca^2+^ to CRT protects a 20–30 kDa CRT domain against trypsin digestion due to a Ca^2+^-dependent conformational change in the protein [31]. In agreement with this report, by SDS-PAGE analysis, the same ~23 kDa CRT fragment and digestion pattern was obtained for the native and recombinant CRTs in the presence of Ca^2+^ ions (Fig. 5 middle panel), whereas addition of EGTA to chelate Ca^2+^ resulted in rapid protein degradation and the absence of a protected fragment (Fig. 5 right panel). We also noticed that CRT proteins showed an additional minor trypsin-resistant fragment of ~50 kDa (Fig. 5 middle panel). A similar ~50 kDa trypsin-resistant fragment for the placental CRT was reported in a previous study [26]. Taken together, native placental and recombinant human CRTs showed similar sensitivity to trypsin digestion indicating a correct folding that has also been previously reported for rabbit CRT expressed in *P. pastoris* [32]. The C-terminal part of the rabbit protein was removed fairly rapidly to give an apparent MW 50-kDa fragment, which was further degraded, leaving a protease-resistant fragment of an apparent 27 kDa [31]. Therefore, limited proteolysis with trypsin indicated that yeast-expressed human CRT was a correctly folded Ca^2+^ binding protein that harbored similar properties to both native human placental CRT [26] and recombinant rabbit CRT expressed in *P. pastoris* [31, 32].

### Functional analysis of the recombinant and native human CRTs

**Fig. 6:**
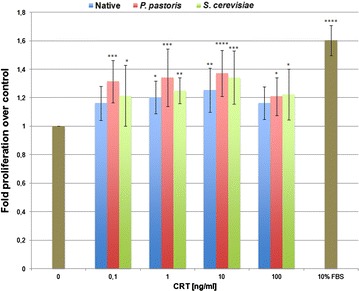
Cellular proliferation induced by native and yeast-derived CRTs. Native CRT isolated from human placenta, and recombinant human CRT purified from yeast *P. pastoris* and *S. cerevisiae* were incubated with human fibroblasts and the assay performed as described in “Methods”. Cellular proliferation was compared to the untreated control of cells incubated in 0.5 % FBS medium without CRT. Experiments were performed in triplicate (n = 6) and *error bars* represent standard error of the mean (*p ≤ 0.05, **p ≤ 0.01, ***p ≤ 0.001 and ****p ≤ 0.0001; Dunnett’s test for statistical significance)

**Fig. 7:**
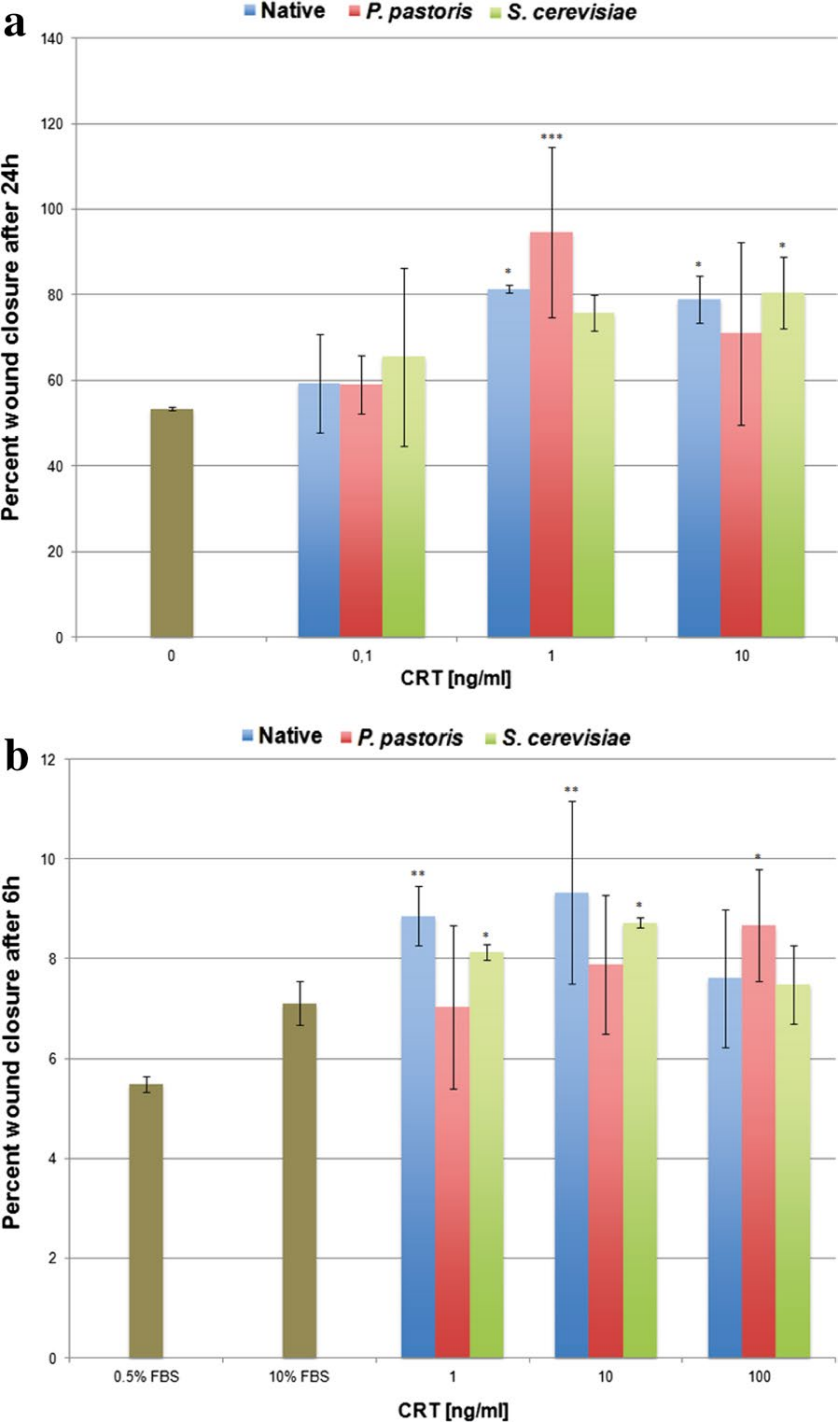
Migration of human dermal fibroblasts induced by native and yeast-derived human CRTs. **a** Primary human dermal fibroblasts were plated, wounded, treated with increasing doses of native placental or recombinant human CRTs (0.1–10 ng/ml) and assayed for wound closure after 24 h, as described in methods. Experiments were performed in triplicate. *Error bars* represent standard deviation (n = 1). **b** Primary human dermal fibroblasts were plated, wounded, treated with increasing concentrations of CRT (1–100 ng/ml), and assayed for wound closure at 6 h. Experiments were performed in triplicate (n = 2). *Error bars* represent standard error of the mean. A Dunnett’s test for statistical significance was performed (*p ≤ 0.05, **p ≤ 0.01, and ***p ≤ 0.001)

It was previously shown that topically applied CRT markedly enhanced the rate and quality of wound healing in porcine and diabetic mouse models [8, 13, 14, 33]. In these studies, CRT was shown to dose-dependently induce wound healing functions of migration, proliferation, and matrix protein induction in human dermal fibroblasts as previously shown in identical in vitro assays [13, 14]. To ensure that CRT secreted from yeast was biologically active, we directly compared effects of native and recombinant CRTs in vitro, for their ability to induce human fibroblast proliferation, using an MTS cell proliferation assay, (Fig. 6) and migration, using a classic in vitro wound healing scratch plate assay (Fig. 7). As shown in Fig. 6, human placental and yeast-derived CRT from both, *P. pastoris* and *S. cerevisiae*, stimulated proliferation at the same peak of activity of 10 ng/ml. At this concentration, native CRT stimulated proliferation by 1.25-fold (p < 0.01) and CRT from *P. pastoris* and *S. cerevisiae* showed a 1.37 (p ≤ 0.0001) and 1.344-fold (p ≤ 0.001) increase, respectively; (untreated control of 0.5 % serum is set at 1.0; n = 6). By comparison, the 10 % FBS positive control stimulated proliferation 1.6-fold over untreated control (p ≤ 0.0001). There was no statistically significant difference among all of the CRTs with all treatment doses. Therefore, we conclude that all CRTs were functionally indistinguishable (Fig. 6). As we have previously shown with receptor mediated responses such as cell proliferation and migration, a peak of activity is obtained, which wanes at higher concentrations of the protein [13, 14]. This can be explained by receptor saturation at concentrations higher than the peak activity shown. In Fig. 6, this downward trend at 100 ng/ml CRT was not statistically significantly different than CRT at 10 ng/ml.

Using the scratch plate method as an in vitro wound healing assay, after 24 h of treatment, 75–95 % wound closure was achieved with all CRT proteins with a peak concentration of 1.0 ng/ml compared to the 0.5 % FBS untreated control, which showed 53 % closure at 24 h (Fig. 7a). Specifically, at this peak concentration, a statistically significant induction of wound closure by native CRT of 81.3 % (p ≤ 0.05) and CRT produced by *P. pastoris* of 94.6 % (p ≤ 0.001) over the untreated control was obtained (n = 1). Whereas statistical significance in induction of wound closure at this concentration was not reached by CRT from *S. cerevisiae*, there was no difference among the three CRTs at 1.0 ng/ml. To test the sensitivity of CRT responsiveness in stimulating migration as measures by wound closure on the scratch, the plates were analyzed at 6 h post-treatment. As shown in Fig. 7b, there was a statistically significant induction of wound closure for native and *S. cerevisiae*-derived CRTs at both 1.0 and 10 ng/ml (n = 2). At the peak concentration of 10 ng/ml CRT, native and *S. cerevisiae*-secreted CRTs stimulated wound closure at 9.3 % (p ≤ 0.01) and 8.7 % (p ≤ 0.05), respectively over the untreated control of 5.5 % closure. There weren’t any statistically significant differences among all CRTs at any dose. Notably, the peak of activity in induction of wound closure was similar with native CRT compared to the two yeast-derived recombinant CRTs suggesting that the functional integrity of yeast-derived CRT was preserved. As described above, the downward trend in activity at 100 ng/ml was obtained previously [13, 14] and likely represents receptor saturation at higher concentrations. Whereas receptor signaling via the LRP1 has been shown for migration [34], which might be operating in the scratch plate assay, the receptor that mediates proliferation is unknown. Earlier we reported that ATPase activity of yeast-secreted human GRP78/BiP protein exceeded the activity of *E. coli*-derived recombinant human BiP threefold [21], whereas yeast-secreted human ERp57 catalyzed the reduction of insulin at a faster rate than analogous recombinant human protein expressed in *E. coli* [22]. However, comparative biological activities to native human proteins were precluded since native protein was not available. The use of native CRT protein isolated from human placenta herein afforded the opportunity to optimally compare the yeast-secreted recombinant protein with purified native human CRT. Similar functional activity of native CRT and recombinant CRT strongly suggest that the synthesized and secreted recombinant CRT should have the same effect on wound healing in vivo, as cellular migration and proliferation are critical functions that enable the wound repair process [13]. As animal studies showed that topical CRT has the potential to be used as an effective therapeutic agent for chronic wounds, including poor healing diabetic wounds [14], the molecular identity between native and yeast-derived CRTs underscores the utility of recombinant CRT secreted from yeast as having clinical and commercial value for wound healing. As the protein consists of the same amino acid sequence, CRT should not be immunogenic as a side effect of treatment.

### High-level secretion of recombinant human CRT by fed-batch fermentation of *P. pastoris*

Development of biopharmaceuticals requires the biosynthesis process to be fully controlled and industrially relevant. *P. pastoris* is known as a highly efficient expression system with well-developed high culture density fermentation protocols. Therefore, we performed synthesis of recombinant secreted human CRT using cultivation of *P. pastoris* in a bioreactor and show the results, which can be adapted for the production of human CRT protein at an industrial scale.

**Fig. 8:**
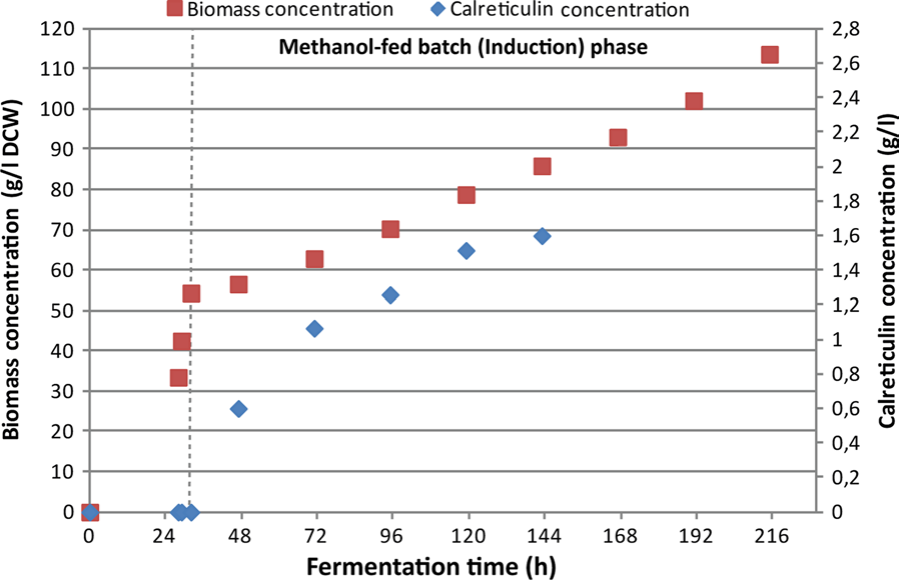
Time-course profile of CRT secretion in high-density *P. pastoris* culture during fed-batch fermentation. *Squares* represent dry cell weight (DCW), *diamonds* show concentration of the secreted CRT in the culture medium. 0–28 h indicate glycerol batch phase, 28–29 h—glycerol fed-batch phase, 29–32 h—transition phase, and 32–215 h—methanol fed-batch phase. Only the amount of intact secreted CRT protein form was calculated

**Fig. 9:**
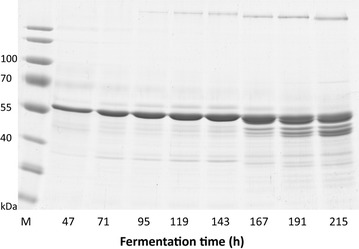
SDS-PAGE analysis of culture supernatant from *P. pastoris* fermentation during the methanol fed-batch stage. Recombinant human CRT was produced as shown in Fig. 8 and described in the text. 2.5 µl of *P. pastoris* culture media, taken at different time points, was loaded onto each lane. *M* prestained protein ladder (ThermoScientific, cat. no. 26616)

A fed-batch cultivation of *P. pastoris* clone CPp9 was carried out in a 5 l bioreactor Biostat A plus using a four-step growth protocol, consisting of glycerol batch, glycerol fed-batch, transition phase and methanol fed-batch phases. The protocol for the fermentation was adapted from Tolner et al. [35] with several modifications to avoid proteolytic degradation of secreted human CRT. The glycerol batch phase was carried out at 28 °C, instead of 30 °C, lasted 28 h, and dry cell weight (DCW) reached 33.5 g/l (Fig. 8). Subsequently, the glycerol batch phase pH of the medium was increased to 7 and the glycerol fed-batch phase was initiated by adding 50 % glycerol and temperature was maintained at 28 °C. Following testing the susceptibility of human CRT to proteases released into the culture broth by *P. pastoris*, pH 7 was found to ensure optimal stability of the protein (data not shown). After 1 h of glycerol-fed batch, DCW reached 42.5 g/l (Fig. 8). The transition phase was started by adding 0.2 % methanol, ramping down the rate of glycerol feeding to 0 ml/l/h, and also decreasing the culture broth temperature to 20 °C. This temperature favoured CRT stability due to decreased release of proteases by yeast cells. After the transition phase, DCW reached 54.4 g/l (Fig. 8) and cells adapted to methanol and lower temperature. 20 °C temperature was maintained throughout the methanol fed-batch phase. Because it was shown [36] that methanol consumption rate at lower temperatures (15 °C) is decreased by 25 %, methanol feed rate during induction phase was decreased by 50 % to 3.8 ml/l/h compared to the feed rate of 7.5 ml/l/h recommended by Tolner et al. [35]. Notably, decreasing the methanol feed rate only by 25 % to 5.6 ml/l/h led to increased proteolytic degradation of human CRT, thus 3.8 ml/l/h methanol feed rate was found to be optimal for biomass and intact protein accumulation (Fig. 8). However, after 135 h induction (167 h fermentation time), secreted human CRT showed signs of degradation and after 183 h induction (215 h fermentation time), degradation was severe (Fig. 9). It was therefore determined that the optimal induction time was 111 h (143 h fermentation time) when secreted human CRT concentration reached 1.6 g/l and DCW was 85.9 g/l (Figs. 8, 9). Specific growth rate on methanol was 0.0042 h^−1^.

### Secretion efficiency of human CRT in yeast

**Fig. 10:**
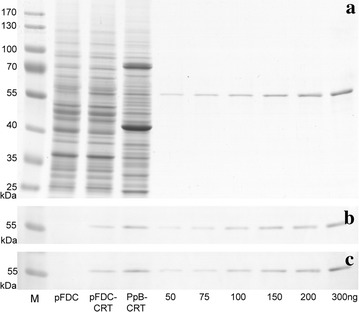
Evaluation of amounts of intracellular human CRT protein in yeast cells. **a** SDS–PAGE of crude yeast lysates and indicated amounts of purified CRT; **b**, **c** two Western blot experiments using monoclonal antibodies against human CRT. *M* prestained protein ladder (ThermoScientific, cat. no. 26616). pFDC, pFDC-CRT and PpB-CRT—crude yeast lysates (10 μg of whole cell protein in *each lane*). pFDC and pFDC-CRT indicate samples from *S. cerevisiae* cells transformed with pFDC vector and pFDC-CRT plasmid, respectively. PpB-CRT sample was taken from fed-batch fermentation of high-density *P. pastoris* culture at 111 h induction time in methanol fed-batch stage (143 h fermentation time). 50, 75, 100, 150, 200, 300—amounts in nanograms of purified *S. cerevisiae*-secreted human CRT protein loaded on gel

To explore the reasons for high-level secretion of human CRT, we determined its secretion efficiency by the same method as previously reported for human GRP78/BiP and ERp57 in yeast [21, 22]. SDS–PAGE analysis of crude lysates from *S. cerevisiae* harbouring pFDC-CRT plasmid revealed a more intense band corresponding to the MW of recombinant human CRT compared to control cells carrying pFDC vector (Fig. 10a, lanes pFDC and pFDC-CRT). The band of comparable intensity and corresponding to the MW of CRT protein was also observed in crude lysate from *P. pastoris* cells, which expressed CRT by high-density fermentation in a bioreactor (Fig. 10a, lane PpB-CRT). Quantitative Western blots using antibodies against human CRT protein showed that intracellular CRT constituted approximately 1.1 % of total cell protein in *S. cerevisiae* (Fig. 10b, c). Evaluation of protein amount according to cell biomass produced from 1 l of *S. cerevisiae* culture revealed that ~30 % of CRT was expressed internally (~25 mg) and ~70 % was secreted into the culture medium (~60–65 mg). Although there was a higher amount of intracellular CRT in *P. pastoris* constituting approximately 2 % of total cell protein (~1.9 and ~2.1 % determined in two different experiments shown in Fig. 10b, c, respectively), the efficiency of CRT secretion reached ~80 % due to the higher amount of secreted protein in the same culture volume (~1.6 g of secreted and ~400 mg of internally expressed CRT). Secretion efficiencies of different human ER chaperones in *S. cerevisiae* can be directly compared, because we used the same expression procedure for CRT, GRP78/BiP and ERp57. Efficiency of CRT secretion (~70 %) was considerably higher than either that of GRP78/BiP (~30 %) [21] or ERp57 (~20 %) [22]. It suggests that CRT is less efficiently retained inside the yeast cells compared to the other two human ER chaperones. Overall higher expression level of CRT could only partially contribute to observed higher CRT levels in *S. cerevisiae* culture media. Total CRT amount was 85–90 mg/l compared to 50–60 mg/l of the other two human ER chaperones (i.e. 1.5–1.8 fold higher, when secreted protein amounts differ four- to fivefold), but there were even lower CRT amounts inside the cells (~25 mg/l vs. 35–50 mg/l for BiP and ERp57). Thus, less efficient intracellular retention of CRT is evident from this data and it seems to be related to intrinsic properties of the protein. It should be noted that all three recombinant human ER chaperones contained an ER-retention/retrieval signal (KDEL for CRT and BiP, or QDEL for ERp57), which did not prevent secretion in yeast. Replacing ER retention signal to yeast preferable ER retention signal HDEL did not suppress the secretion of these three proteins (our unpublished data). Moreover, overload of the yeast ER retrieval machinery as the reason for secretion of human ER chaperones can also be omitted, because overexpression of yeast Kar2 protein with native HDEL ER retrieval sequence using the same pFDC vector did not lead to the secretion of this protein [21]. These results indicate that the mechanism(s) involved are currently elusive and deserve further investigation. We propose that one of the reasons might be limited accessibility of the ER retrieval signals of human ER chaperones in the yeast cells. For example, ER calcium depletion was shown to induce CRT secretion [18], whereas free Ca^2+^ in the lumen of the yeast ER is 10–100 times lower than that of mammalian ER [37, 38]. Such distinctly different characteristics may result in a CRT conformation, which limits accessibility of the KDEL retrieval signal and favours protein secretion outside of the yeast cells. Nevertheless, this issue requires additional studies.

### Significance and applicability of the results

With a growing demand for human CRT biotherapeutics, we introduce yeast *S. cerevisiae* and *P. pastoris* as excellent hosts for easily purified, high-level production of human CRT. Here we demonstrated that the native signal sequence of human CRT precursor is correctly cleaved and directs secretion in yeast cells. High-level secretion of human CRT using fed-batch fermentation of high-density *P. pastoris* culture in a bioreactor allowed efficient production of secreted CRT protein in grams per liter scale. To the best of our knowledge, this is one of the highest secretion levels achieved using native human protein signal sequences in yeast. Comparable results after optimization were achieved only with human serum albumin (HSA) [39]. Our data suggest that molecular integrity and functional activity of yeast-secreted recombinant CRT is identical to native CRT protein from human placenta. From these results, we can extrapolate that CRT secreted by yeast should be inherently biologically active in any functional in vitro and in vivo acitivity (harboured by native CRT) with applicability to the development of new biopharmaceuticals such as has been shown for normal and diabetic wound healing [13, 14]. Moreover, it was recently shown that this same yeast-secreted CRT can also be used as a cancer treatment adjuvant in combination with photodynamic therapy [40]. Yeast-secreted recombinant protein may be advantageous in such applications, because it corresponds to the native analogue insofar as possible. Furthermore, yeast-derived heterologous proteins are free of toxic contaminations and are excellent tools for developing biopharmaceuticals, because *S. cerevisiae* is acknowledged as a GRAS (generally regarded as safe) organism. Therefore, secretory expression of native recombinant human CRT in yeast could be exploited for efficient and safe production of potential therapeutic agents.

## Conclusions

Our results show that yeast is an excellent host for efficient and industrial scale production of native recombinant human CRT. Yeast cells are able to recognize and correctly process the native signal sequence of human CRT precursor. Yeast secretes mature CRT protein into the culture medium, instead of retaining the protein in the ER. Unusual high-level secretion enabled CRT production in grams per liter scale with a simple and cost-effective purification scheme. Recombinant CRT secreted by yeast appears seemingly identical to the native protein from human placenta with respect to its molecular integrity and functional activity in vitro. The yeast expression and purification systems for human CRT offer a new way of producing large quantities of human protein for therapeutic application.

## Methods

### Plasmids, yeast strains, transformation and selection of transformants

DNA manipulations were performed according to standard procedures [41], bacterial recombinants were screened in *Escherichia coli* DH5αF′ cells. A cDNA encoding full-length human calreticulin precursor (GenBank Acc. no. M84739) was amplified from a commercial human adult liver cDNA library (Clontech) by PCR using specific oligonucleotide primers CRTF (gta **tct aga** aca atg ctg cta tcc gtg ccg ttg) and CRTR (cag **tct aga** cta cag ctc gtc ctt ggc ctg), digested with restriction endonuclease (RE) XbaI (recognition sites in sequences of primers are indicated in bold) and cloned into yeast expression vectors pFDC [20] and pPIC3.5K (Invitrogen) into RE sites XbaI and AvrII under the control of *S. cerevisiaePGK1* or *P. pastoris AOX1* promoters, respectively. Cloned *CRT* gene coding sequence (beginning from start codon ATG and ending with STOP codon TAG) was verified by DNA sequencing and generated plasmids pFDC-CRT and pPIC3.5K-CRT were used for transformation of yeast *S. cerevisiae* strain AH22 (MATa *leu2 his4*) and *P. pastoris* strain GS115 (*his4*), respectively. *S. cerevisiae* transformants were selected by resistance to formaldehyde [21] and harboured multicopy autonomously replicating plasmid pFDC-CRT, whereas multicopy *P. pastoris* transformants were selected by resistance to G418 [42] and Mut^+^ clone CPp9 with the most efficient secretion of CRT protein was chosen for further experiments. Both yeasts were used for expression of the full length CRT protein precursor including the native N-terminal signal peptide.

### Protein expression in shake-flask cultivations and purification of recombinant CRT from yeast culture media

*Saccharomyces cerevisiae* cells harbouring plasmid pFDC-CRT were grown for 36 h in YEPD (yeast extract 1 %, peptone 2 %, dextrose 2 %) medium. *P. pastoris* transformants with multicopy integrations of expression vector pPIC3.5K-CRT were initially grown in Ygly medium containing glycerol as carbon source (yeast extract 1 %, peptone 2 %, glycerol 1 %, biotin 2 × 10^−5^ %) for 24 h up to OD of 18–20, and culture medium was changed to Ymet containing methanol (yeast extract 1 %, peptone 2 %, methanol 1 %, biotin 2 × 10^−5^ %) for induction of *CRT* cDNA expression. Then flasks were further incubated in the shaker at 30 °C, 220 rpm for 120 h. 1 % of methanol was added every 8 h to maintain protein expression. Both *P. pastoris* and *S. cerevisiae* cultures were centrifuged at 10,000×*g* for 10 min at 4 °C. Supernatants were collected and stored on ice. Protocol for CRT purification from culture media was the same for both yeasts.

Yeast culture medium was microfiltered through 1.6 µM (Sartorius Stedim Biotech, cat. no. FT-3-1101-047), 0.45 µM (Sartorius Stedim Biotech, cat. no. 15406-47) and 0.2 µM (Sartorius Stedim Biotech, cat. no. 15407-47-MIN) filters using a Pressure Filter Holder (Sartorius Stedim Biotech, cat. no. 16249) and a vacuum pump. After microfiltration, proteins from the culture medium were concentrated and transferred into the binding buffer (20 mM l-histidine, 100 mM NaCl, pH 5.5) through tangential ultrafiltration using cassettes with 100 kDa cut-off membranes (Sartorius Stedim Biotech, cat. no. VF20P4) and a peristaltic pump. Further, proteins were loaded onto the column packed with Q Sepharose FastFlow resin (GE Healthcare, cat. no. 17-0510-10) equilibrated in the same buffer. The column was washed with 5 volumes of binding buffer and bound proteins were eluted with a NaCl concentration gradient (100–500 mM). CRT was eluted in a single peak with approximately 250 mM NaCl. Elution fractions containing purified recombinant protein were pooled and buffer was exchanged to CRT storage buffer (20 mM Tris–HCl, 150 mM NaCl, 3 mM CaCl_2_, pH 7.5) using Sephadex G25 column. Protein was stored frozen at −70 °C.

### Purification of native CRT from human placenta

Human placentas were donated for research with written consent from women who had given birth to healthy children at Rigshospitalet, Copenhagen. At the Statens Serum Institut they were used anonymously for purification of CRT. Human placental CRT was purified as described previously [26]. Briefly, a placenta was homogenized twice in 20 mM BisTris, 1 mM CaCl_2_, pH 7.5 with protease inhibitors (Complete) and with intermittent centrifugations. The residue was then extracted twice in 20 mM BisTris, 1 mM CaCl_2_, pH 7.5, 1 % Triton X-114 to release ER luminal proteins. Membrane proteins were removed by phase separation at 37 °C and large proteins removed from the water phase by ammonium sulphate precipitation (337 g/l) overnight at 5 °C. The supernatant was ultradiafiltrated against 20 mM Tris, 1 mM CaCl_2_, pH 7.5 and chromatographed on a Q Sepharose column equilibrated in the same buffer and eluted with a linear NaCl gradient. CRT-containing fractions were pooled and concentrated to 1 mg/ml in 20 mM Tris/HCl, pH 7.5 using a Centriprep filter. Purified placental CRT was stored frozen at −20 °C.

### Partial proteolysis of CRT with trypsin in the presence of calcium

Partial digestion of CRT with trypsin was performed according to previously described procedures [26, 31]. Purified yeast-secreted CRT was diluted to 1 mg/ml concentration in storage buffer containing 3 mM CaCl_2_. To avoid influence of different storage buffers, native placental CRT was also transferred to storage buffer with 3 mM CaCl_2_ as for recombinant CRTs, and diluted to the same concentration. Digestion was performed at 37 °C in 50 µl volume by adding 1 μl of 0.5 mg/ml trypsin [ratio of CRT:trypsin was 100:1 (w/w)]. In a control tube, calcium was removed by adding EGTA to 5 mM concentration. The reaction was stopped after 60 min, by adding 1 mM PMSF. The samples were boiled, loaded onto the gels, and SDS-PAGE was performed.

### Function of yeast-derived calreticulin: cellular migration and proliferation assays

*Cell proliferation* An in vitro assay for cellular proliferation was performed as previously described [13, 14]. Primary low passage human dermal foreskin fibroblasts (CCD 1070SK; American Type Culture Collection, Manassas, VA, USA), grown in complete Eagle’s minimal essential medium (MEM; Gibco/Life Technologies, Carlsbad, CA, USA) containing 10 % fetal bovine serum (FBS), 2 mM glutamine (Mediatech, Manassas, VA, USA), 1.0 mM sodium pyruvate (Gibco) and antibiotics, were seeded at 2 × 10^3^ cells per well in 96 well plates and grown to 50 % confluency (24 h). Subsequently, the cells were synchronized in media containing 0.5 % FBS for 24 h and treated with increasing concentrations of CRT (0–100 ng/ml) from placenta, or *S. cerevisiae* or *P. pastoris* for 72 h. CRT protein concentrations were determined by microBCA assay to standardize that equal amounts of each CRT were used. The assay was performed in triplicate for each test parameter. Media with 0.5 % FBS and 10 % FBS served as a negative and positive controls, respectively. Proliferation was determined by an MTS assay (CellTiter96 assay, G3580, Promega, Madison, WI, USA). Cells were incubated in MTS solution for 1 h and absorbance was measured at 490 nm (BioRad ELISA reader). The data are expressed as fold proliferation over untreated control (n = 6 experiments).

*Cell migration* The effect of native human placental and yeast-derived human CRT on cellular migration was assessed by the classic in vitro wound healing scratch plate assay, as described [13, 14]. Human dermal fibroblasts in complete MEM containing 10 % FBS were seeded at 2 × 10^4^ cells per well in 24 well plates and grown to 70–80 % confluency. The cells were switched to media containing 0.5 % FBS for 24 h, and then wounds were created down the center of each well by scratching with a 200 µl pipette tip, and the loose cells removed by washing with PBS. The edges of the wounds were denoted with a fine-tipped marker on the underside of the plate. The cells were treated for 6 or 24 h with increasing concentrations of CRT protein in 0.5 % FBS media. 0.5 and 10 % FBS served as negative and positive controls, respectively. Migration was terminated by incubating the cells with 0.025 % Coomassie blue in 10 % acetic acid, 45 % methanol for 10 min followed by washing twice with PBS. The plates were viewed under an inverted light microscope (Axiovert s-100; Zeiss, Thornwood, NY, USA) and images captured and cell migration calculated by measuring the area (pixels) not occupied by cells (i.e., open area remaining) to the area of the original scratch at time 0 using Metamorph software version 7.1.3.0 (Molecular Probes, Eugene OR, USA). The plates were normalized for cell density at a part of the plate distal to the scratch. The data are expressed as per cent wound closure (0 time compared to 6 or 24 h later) and the averages for each test parameter subjected to analysis of variance (ANOVA). Statistical significance for both proliferation and wound closure assays were performed using GraphPad Prism software (version 6). A two-way ANOVA followed by a Dunnett post hoc test by comparing each treatment value with 0.5 % FBS as the untreated control; statistical significance was (*p* < 0.05). The experiments were performed in triplicate (n = 2 experiments at 6 h and n = 1 at 24 h).

### High cell density fed-batch fermentation of recombinant *P. pastoris*

For this study, selected Mut^+^ phenotype *P. pastoris* GS115 transformant strain CPp9 carrying multiple copies of human *CRT* gene under the control of *AOX1* promoter, was used. A 200 ml culture for inoculation of bioreactor was prepared in two stages as described previously [35]. First, a 20-ml primary seed YEPD medium was prepared using 200 μl of the user seed lot which was grown at 28 °C in an orbital shaker (New Brunswick Innova 40R) at 220 rpm for 24 h. This primary seed medium was used to inoculate a 200 ml secondary seed medium, containing fermentation media, to 0.1 OD that was grown for 16–18 h to 4–6 OD under the same conditions, to serve as inoculum for the bioreactor culture. Cultures were grown in baffled flasks whose volumes were 5× the culture volume to permit adequate aeration.

High-cell density fed-batch cultivation was performed as described previously [35] with several exceptions. Cultivation was carried out in a 5 l BIOSTAT-A plus (Sartorius Stedim Biotech) bioreactor interfaced with MFCS/DA software for data acquisition and control. A 200 ml inoculum, prepared as described above, was transferred to the bioreactor containing 1.8 l fermentation media. The fermentation media contained per liter: glycerol, 63 g; potassium sulfate, 14.67 g; calcium sulfate, 0.9 g; magnesium sulfate hepta-hydrate, 11.67 g; trace metal solution (PTM1), 4 ml; ammonium sulfate, 9 g; hexametaphosphate, 25 g. The PTM1 solution contained per liter: sodium iodide, 0.08 g; manganese sulfate mono-hydrate, 3.0 g; sodium molybdate di-hydrate, 0.2 g; boric acid, 0.02 g; zinc chloride, 20.0 g; ferric sulfate hepta-hydrate, 65.0 g; cupric sulfate penta-hydrate, 6.0 g; biotin, 0.2 g; and sulfuric acid, 5.0 ml. 0.2 ml of antifoam (Antifoam 204, SigmaAldrich) per 1 l media was added manually before inoculation to control foaming in the bioreactor. Aeration rate of 1 vvm was constant throughout the whole process. The dissolved oxygen (DO) throughout the whole process was controlled at 30 % saturation using an automatic DO control by agitation cascade between 410 and 750 rpm and oxygen supplementation. When a maximum 750 rpm was reached, pure oxygen was supplied through a gas blender to control dissolved oxygen at 30 % saturation.

Temperature during glycerol batch phase was maintained at 28 °C and pH at 5.0 with 28 % (v/v) NH_4_OH. After consumption of glycerol, indicated by an increase of the DO concentration, glycerol-fed batch phase was initiated. Temperature during glycerol-fed batch phase was maintained at 28 °C and pH at 7.0. 50 % glycerol (v/v) with PTM1 was fed at 20 ml/l/h for 1 h before being ramped down to 0 ml/l/h at a uniform rate over a 3-h period. The ramping down of glycerol marked the beginning of the transition phase and 2 ml/l of methanol was added to the bioreactor to allow the cells to adjust to methanol. Temperature during transition phase was ramped down to 20 °C and pH was maintained at 7.0. After transition phase methanol-fed batch phase (production of recombinant CRT) was initiated by the addition of methanol at a 2 ml/l/h rate which was increased to 3.8 ml/l/h at a uniform rate over a 6 h period and 3.8 ml/l/h methanol feed rate was maintained during whole methanol-fed batch. Temperature during methanol-fed batch phase was maintained at 20 °C and pH at 7.0. For DCW determination, cell pellets were washed once with distilled water and incubated in an open tube to a constant weight at 80 °C.

### Miscellaneous

Tryptic peptide mass fingerprinting was carried out at the Proteomics Center in the Institute of Biochemistry of Vilnius University (Lithuania). *S. cerevisiae*-secreted CRT was analyzed by MALDI-TOF/TOF tandem MS/MS (mass spectrometry) as described previously [43]. Trypsin digestion of *P. pastoris*-secreted CRT was done according to a modified FASP protocol as described by Wisniewski et al. [44]. The nano-LC was coupled online with an HDMS Synapt G2 mass spectrometer (Waters Corporation, UK). LC–MS data were collected using data independent acquisition (DIA) mode MS^E^ in combination with online ion mobility separation.

N-terminal sequencing of yeast-secreted human CRT protein by Edman degradation was performed by AltaBioscience.

The molecular masses of proteins were measured by ESI–MS using an Agilent Q-TOF 6520 mass spectrometer.

Native PAGE was performed exactly as described previously [21], whereas blue native PAGE was carried out according to Niepmann and Zheng [45].

Protein concentrations were determined by Roti-Nanoquant Protein-assay (Carl Roth Gmbh., cat. no. K880) using BSA for calibration curve.

Densitometric analysis of SDS-PAGE gels scanned with ImageScanner III (GE Healthcare) was performed with ImageQuant TL (GE Healthcare) software using default settings. For quantitative analysis of secreted CRT, the culture medium was separated from cells by centrifugation, directly mixed with equal amount of 2× SDS-PAGE sample buffer [46], boiled and loaded onto gel lanes. Amounts of secreted CRT in culture medium were determined by comparison to a range of known amounts of BSA (Fermentas product #B14 was used) in a linear dynamic range (R^2^ > 0.99) of Coomassie-stained protein bands in SDS-PAA gels. To determine CRT purity in crude yeast culture media, total secreted yeast proteins were concentrated by precipitation and yeast protein impurities were evaluated by SDS-PAGE.

Precipitation of proteins from yeast culture medium for SDS-PAGE analysis was performed based on a defined methanol-chloroform-water mixture, as described earlier [47].

Mouse monoclonal anti-CRT antibody [FMC 75] was purchased from Abcam (cat. no. ab22683-100).

## Additional files

10.1186/s12934-015-0356-8 Different secretion levels of human ER chaperones in yeast. SDS-PAGE (A) and Western blot (B) analysis of 40× concentrated culture media of yeast *S. cerevisiae* AH22 cells transformed with empty plasmid pFDC (lane 1) or producing human chaperones BiP, CRT and ERp57 (lanes 2, 3 and 4, respectively). Anti-GRP78 BiP antibody (ab21685), mAb anti-CRT FMC 75 and ERp57 (MaP.ERp57) antibody (sc-23886) were used in Western blots to detect recombinant BiP, CRT and ERp57, respectively.
10.1186/s12934-015-0356-8 Elution profiles of recombinant CRT in anion-exchange chromatography. Separation of CRT from *S. cerevisiae* (A and B) was performed on a pre-packed 5 ml Q-Sepharose column (GE LifeSciences cat. no. 17-5156-01), equilibrated with 20 mM l-His, pH 5.5, 100 mM NaCl buffer, in 15 column volumes linear gradient from 100 mM to 500 mM NaCl. Separation of CRT from *P. pastoris* (C and D) was performed on a 15 ml Q-Sepharose sorbent (GE LifeSciences cat. no. 17-0510-10) packed into the XK 16/20 column (GE Lifesciences, cat. no. 28-9889-37), equilibrated with 20 mM l-His, pH 5.5, 100 mM NaCl buffer, in 10 column volumes linear gradient from 100 to 500 mM NaCl. Elution profiles (A and C) and SDS-PAGE gels of corresponding fractions (B and D) are shown. Dashed lines define fractions that were pooled and used for further experiments with purified protein.
10.1186/s12934-015-0356-8 Western blotting analysis of recombinant and native human CRTs. The same samples were analysed by SDS-PAGE (A) and Western blot (B) using monoclonal antibodies against human CRT. M—prestained protein ladders. pFDC and pFDC-CRT—10× concentrated culture media samples from *S. cerevisiae* transformed with pFDC vector and pFDC-CRT plasmid, respectively. Sc, Pp and Hn—purified CRT from *S. cerevisiae*, *P. pastoris* and human placenta, respectively. Crude lysate of *S. cerevisiae* cells transformed with pFDC vector was used as negative control for Western blot. Bl—blank lanes.
10.1186/s12934-015-0356-8 Comparison of different CRT preparations from *S. cerevisiae*. Four different preparations (A, B, C, D) of purified human CRT from *S. cerevisiae* were analysed by SDS-PAGE (at the left) and Western blotting (at the right) using monoclonal antibodies against human CRT. M—prestained protein ladder (ThermoScientific, cat. no. 26616).
10.1186/s12934-015-0356-8 Native PAGE of human placental and recombinant CRTs from the same storage buffer. Human placental CRT was transferred to the same buffer used for storage of recombinant CRTs (20 mM Tris–HCl, 150 mM NaCl, 3 mM CaCl_2_, pH 7.5) and all three CRTs were analysed by electrophoresis under native conditions. Blue native PAGE is shown above and native PAGE below, all references are the same as in Fig. 2 legend.
