# Supplementary figures and images for: High-level secretion of native recombinant human calreticulin in yeast
Source: Microb Cell Fact. 2015 Oct 15;14:165. doi: 10.1186/s12934-015-0356-8 (PMC4608220; doi:10.1186/s12934-015-0356-8)

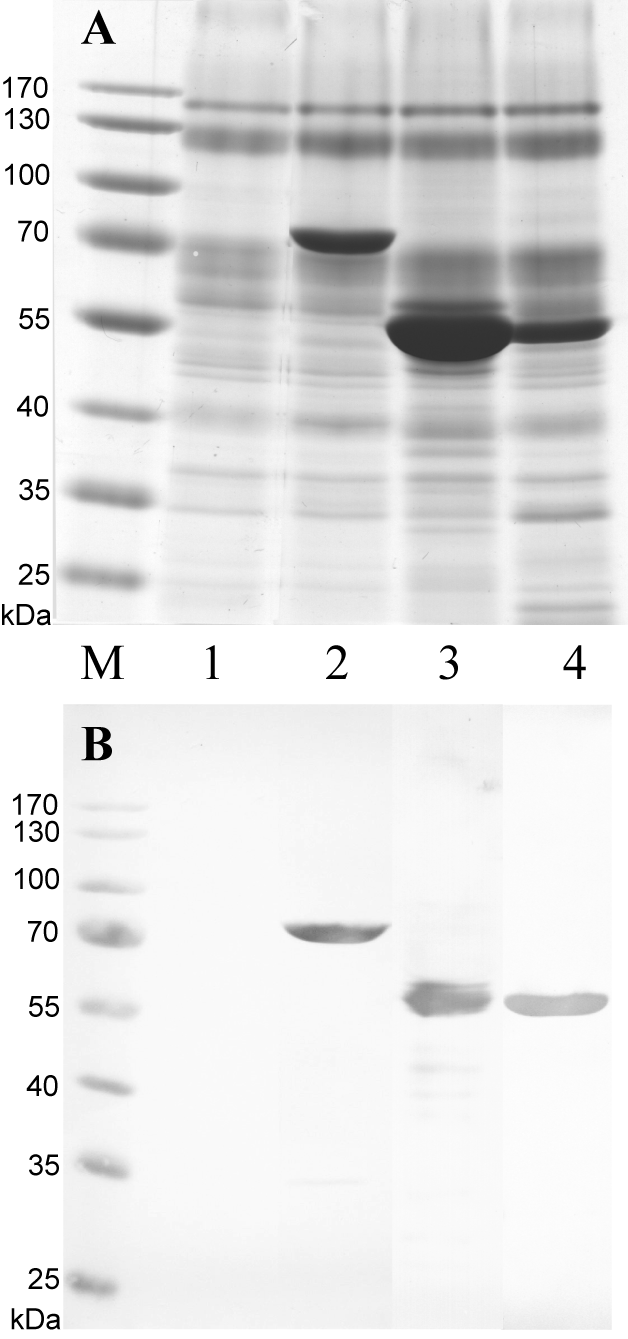

Supplement: Supplementary file 1 — 10.1186/s12934-015-0356-8 Different secretion levels of human ER chaperones in yeast. SDS-PAGE (A) and Western blot (B) analysis of 40× concentrated culture media of yeast S. cerevisiae AH22 cells transformed with empty plasmid pFDC (lane 1) or producing human chaperones BiP, CRT and ERp57 (lanes 2, 3 and 4, respectively). Anti-GRP78 BiP antibody (ab21685), mAb anti-CRT FMC 75 and ERp57 (MaP.ERp57) antibody (sc-23886) were used in Western blots to detect recombinant BiP, CRT and ERp57, respectively. [file 12934_2015_356_MOESM1_ESM.tif]

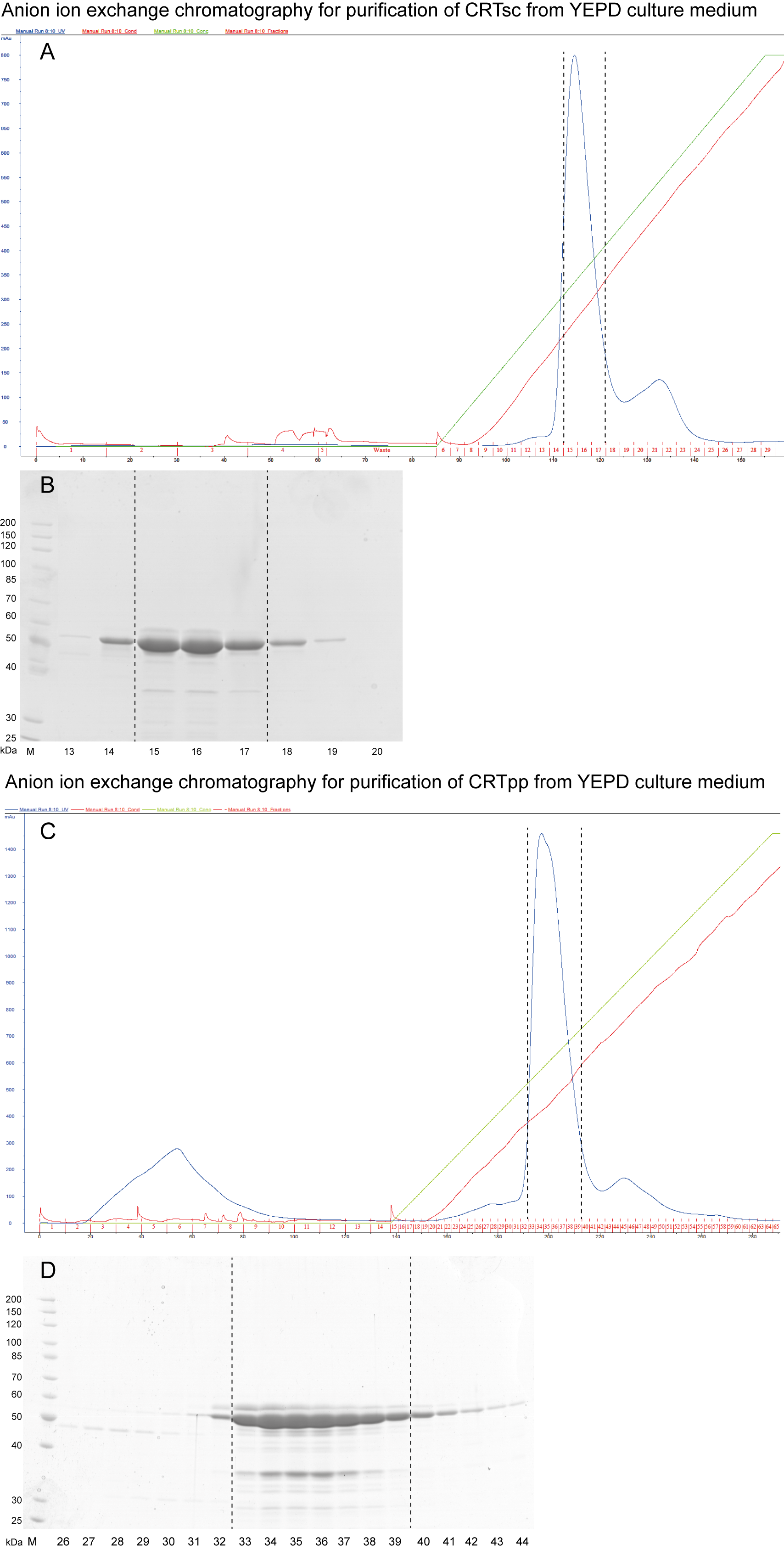

Supplement: Supplementary file 2 — 10.1186/s12934-015-0356-8 Elution profiles of recombinant CRT in anion-exchange chromatography. Separation of CRT from S. cerevisiae (A and B) was performed on a pre-packed 5 ml Q-Sepharose column (GE LifeSciences cat. no. 17-5156-01), equilibrated with 20 mM l-His, pH 5.5, 100 mM NaCl buffer, in 15 column volumes linear gradient from 100 mM to 500 mM NaCl. Separation of CRT from P. pastoris (C and D) was performed on a 15 ml Q-Sepharose sorbent (GE LifeSciences cat. no. 17-0510-10) packed into the XK 16/20 column (GE Lifesciences, cat. no. 28-9889-37), equilibrated with 20 mM l-His, pH 5.5, 100 mM NaCl buffer, in 10 column volumes linear gradient from 100 to 500 mM NaCl. Elution profiles (A and C) and SDS-PAGE gels of corresponding fractions (B and D) are shown. Dashed lines define fractions that were pooled and used for further experiments with purified protein. [file 12934_2015_356_MOESM2_ESM.tif]

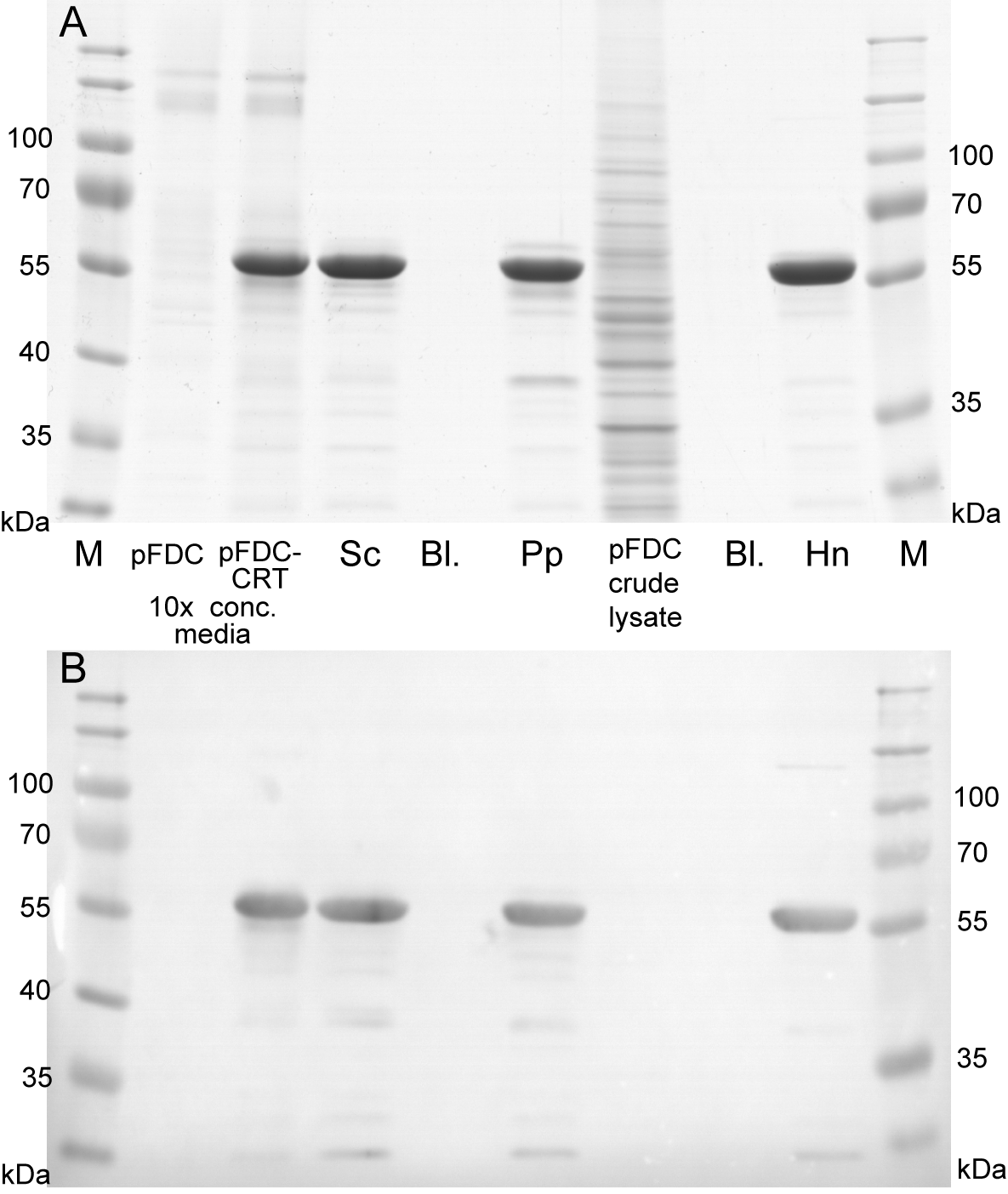

Supplement: Supplementary file 3 — 10.1186/s12934-015-0356-8 Western blotting analysis of recombinant and native human CRTs. The same samples were analysed by SDS-PAGE (A) and Western blot (B) using monoclonal antibodies against human CRT. M—prestained protein ladders. pFDC and pFDC-CRT—10× concentrated culture media samples from S. cerevisiae transformed with pFDC vector and pFDC-CRT plasmid, respectively. Sc, Pp and Hn—purified CRT from S. cerevisiae, P. pastoris and human placenta, respectively. Crude lysate of S. cerevisiae cells transformed with pFDC vector was used as negative control for Western blot. Bl—blank lanes. [file 12934_2015_356_MOESM3_ESM.tif]

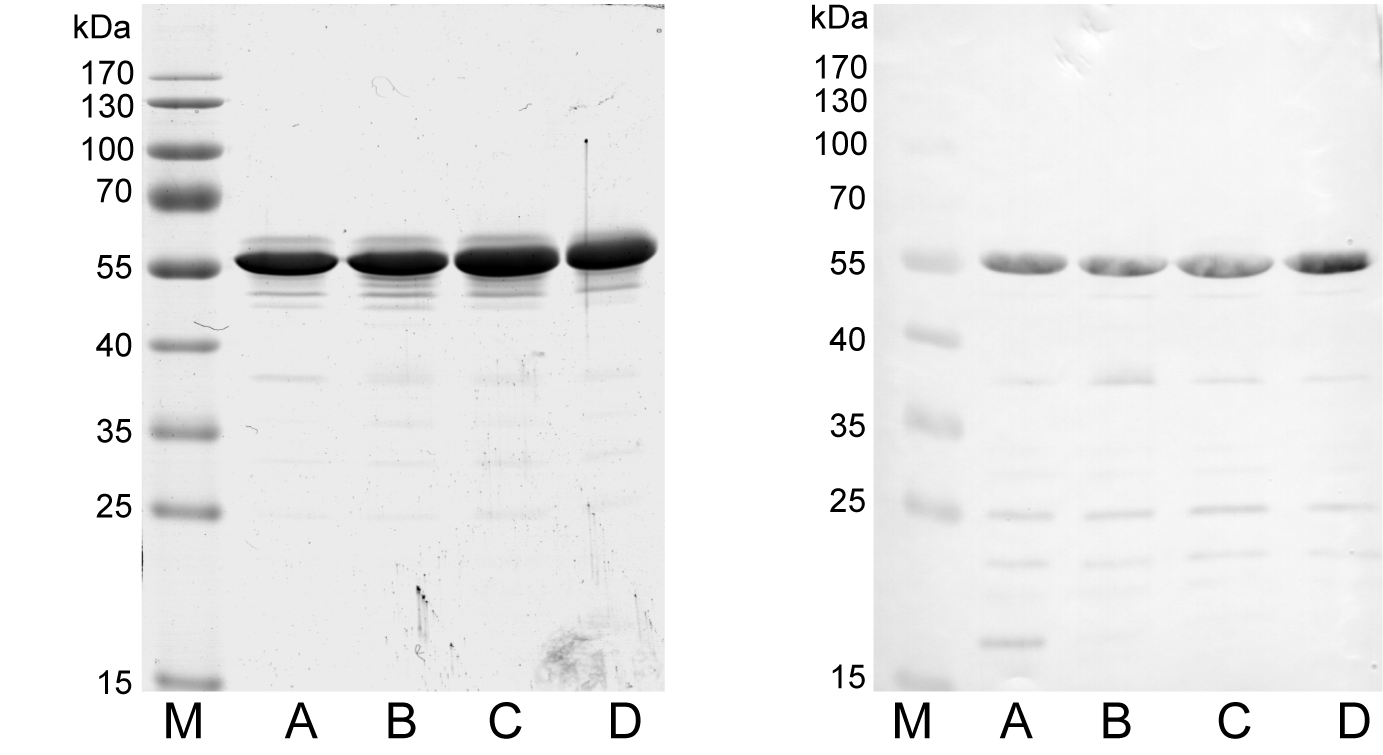

Supplement: Supplementary file 4 — 10.1186/s12934-015-0356-8 Comparison of different CRT preparations from S. cerevisiae. Four different preparations (A, B, C, D) of purified human CRT from S. cerevisiae were analysed by SDS-PAGE (at the left) and Western blotting (at the right) using monoclonal antibodies against human CRT. M—prestained protein ladder (ThermoScientific, cat. no. 26616). [file 12934_2015_356_MOESM4_ESM.tif]

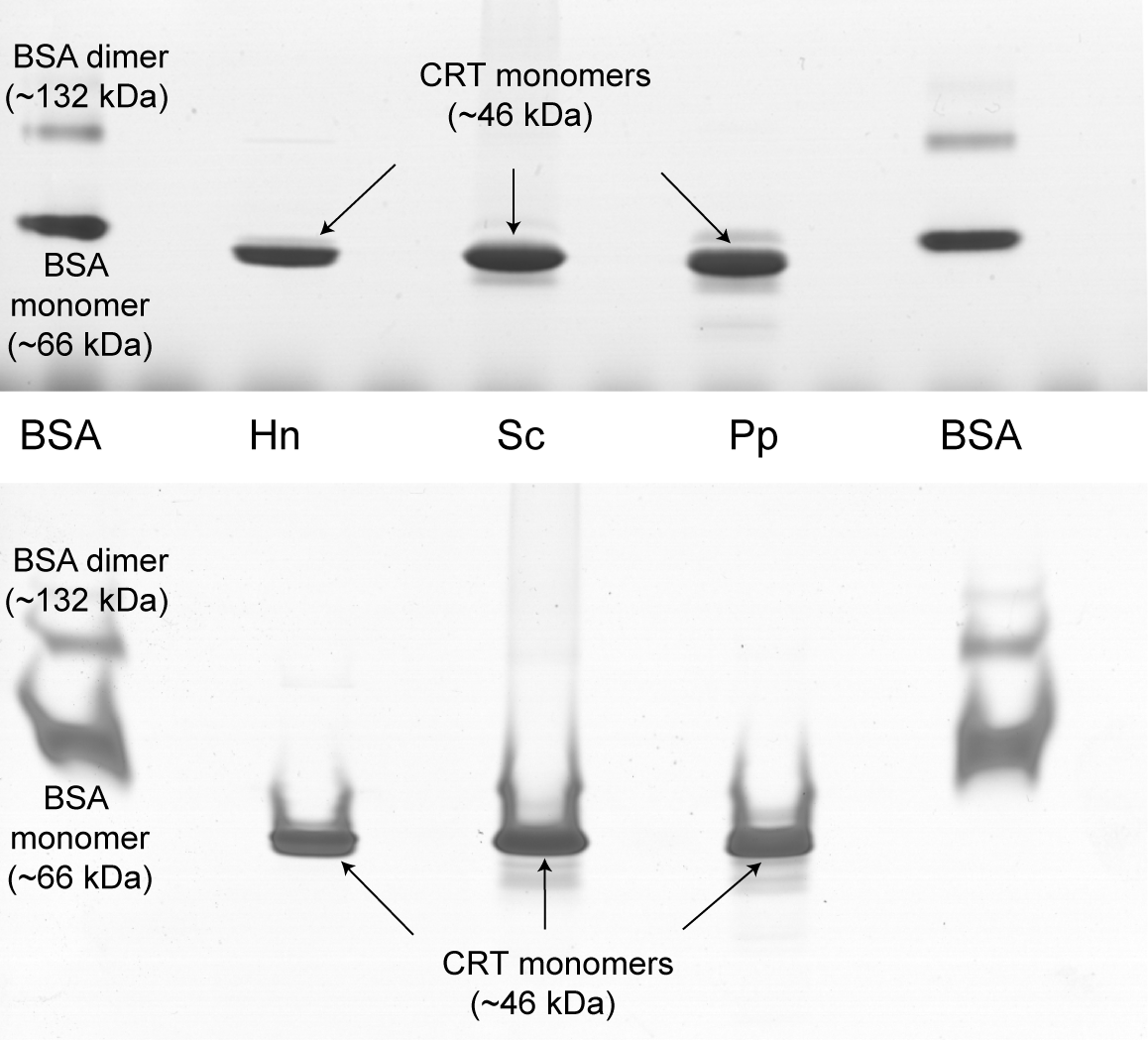

Supplement: Supplementary file 5 — 10.1186/s12934-015-0356-8 Native PAGE of human placental and recombinant CRTs from the same storage buffer. Human placental CRT was transferred to the same buffer used for storage of recombinant CRTs (20 mM Tris–HCl, 150 mM NaCl, 3 mM CaCl2, pH 7.5) and all three CRTs were analysed by electrophoresis under native conditions. Blue native PAGE is shown above and native PAGE below, all references are the same as in Fig. 2 legend. [file 12934_2015_356_MOESM5_ESM.tif]
